# Update on the Use of Nanocarriers and Drug Delivery Systems and Future Directions in Cervical Cancer

**DOI:** 10.1155/2022/1636908

**Published:** 2022-05-04

**Authors:** Loredana Maria Himiniuc, Bogdan Florin Toma, Razvan Popovici, Ana Maria Grigore, Alexandru Hamod, Constantin Volovat, Simona Volovat, Irina Nica, Decebal Vasincu, Maricel Agop, Mihaela Tirnovanu, Lacramioara Ochiuz, Anca Negura, Mihaela Grigore

**Affiliations:** ^1^”Grigore T. Popa” University of Medicine and Pharmacy, Iasi 700115, Romania; ^2^Department of Obstetrics and Gynecology, “Grigore T. Popa” University of Medicine and Pharmacy, Iasi 700115, Romania; ^3^Regional Institute of Oncology, 700483 Iasi, Romania; ^4^Center of Oncology Euroclinic, 700110 Iasi, Romania; ^5^“Grigore T. Popa” University of Medicine and Pharmacy, Department of Medical Oncology Radiotherapy, 700115 Iași, Romania; ^6^Department of Odontology, Periodontics and Fixed Restoration, “Grigore T. Popa” University of Medicine and Pharmacy, Iasi 700115, Romania; ^7^Department of Dental and Oro-Maxillo-Facial Surgery, “Grigore T. Popa” University of Medicine and Pharmacy, Iasi 700115, Romania; ^8^Department of Physics, “Gheorghe Asachi” Technical University of Iasi, Iasi 700050, Romania; ^9^Romanian Scientists Academy, Bucharest 050094, Romania; ^10^Department of Pharmaceutical and Biotechnological Drug Industry, “Grigore T. Popa” University of Medicine and Pharmacy, Iasi 700115, Romania; ^11^Oncogenetics Department, “Grigore T. Popa” University of Medicine and Pharmacy, 700115 Iași, Romania; ^12^Biology Department, “Alexandru Ioan Cuza” University, 700506 Iaşi, Romania

## Abstract

Cervical cancer represents a major health problem among females due to its increased mortality rate. The conventional therapies are very aggressive and unsatisfactory when it comes to survival rate, especially in terminal stages, which requires the development of new treatment alternatives. With the use of nanotechnology, various chemotherapeutic drugs can be transported via nanocarriers directly to cervical cancerous cells, thus skipping the hepatic first-pass effect and decreasing the rate of chemotherapy side effects. This review comprises various drug delivery systems that were applied in cervical cancer, such as lipid-based nanocarriers, polymeric and dendrimeric nanoparticles, carbon-based nanoparticles, metallic nanoparticles, inorganic nanoparticles, micellar nanocarriers, and protein and polysaccharide nanoparticles. Nanoparticles have a great therapeutic potential by increasing the pharmacological activity, drug solubility, and bioavailability. Through their mechanisms, they highly increase the toxicity in the targeted cervical tumor cells or tissues by linking to specific ligands. In addition, a nondifferentiable model is proposed through holographic implementation in the dynamics of drug delivery dynamics. As any hologram functions as a deep learning process, the artificial intelligence can be proposed as a new analyzing method in cervical cancer.

## 1. Introduction

Cervical cancer (CC) represents nowadays a serious medical challenge despite the early efforts to diagnosis and treatment. One of the most common causes of precancerous cervical lesions represents the persistent infection of the cervix with “high risk” genotypes of *Human papillomavirus* (HPV). If the continuous infection is not early treated in time, it can determine invasive CC [1]. Other factors such as immunosuppression, parity, smoking, and use of oral contraceptives may also contribute to CC promotion [2]. Cervical cancer-related mortality represents worldwide the fourth leading cause and the most often diagnosed cancer in females among 23 countries. In 2020, the global estimated number of new CC cases reached 604,000. On the other hand, 342,000 females died because of CC and most of them represented women from low- and middle-income countries. Still, available large-scale screening methods, the raise of socioeconomic status in different regions, and the reduction of HPV persistent infection risks within population have decreased over the last decades the incidence and mortality rates related to CC [3].

Since it was found that HPV is necessary but not sufficient to develop CC, more than 100 types of HPV have been studied, and a significant number of them have been incriminated to play an important role in the cancer pathogenesis [3]. *Human papillomavirus* is a nonenveloped double-stranded DNA virus, which present a high affinity to the mucosa or the skin. The high-risk HPV (Hr-HPV) genotypes, such as HPV 16, 18, 31, 33, 35, 39, 45, 51, 52, 56, 58, 59, 66, 68, and 70 are associated with mucosal infection and may slowly progress into intraepithelial neoplasia, and further, into invasive cancer. Contrariwise, the low-risk HPV (Lr-HPV) types such as 6, 11, 42, 43, and 44 often determine benign cutaneous lesions like warts and are not usually associated with malignant lesions. The mature HPV particle has a size of 50-60 nm and consists of around 8000 bp genome that comprises an icosahedral capsid and various core genes involved in replication and transcription (E1, E2), packaging (L1, L2), or which have various roles such as stimulating the cell cycle entrance, viral immunity avoidance or viral release and transmission (E4, E5, E6, E7) [4, 5].

The development of HPV-infected epithelial cells to invasive cancer is a lasting progress linked to the assembling of the DNA changes inside the host cell genes [4]. *Human papillomavirus* cycle starts with the microtraumas of the basal layer and its HPV infection that impair the epithelial barrier. When the epithelial cells start their differentiation, an increased number of viral copies replicates and expresses L1 and L2 core genes, creating new virions that are launched from the epithelial layer. For persistent HPV infection, the virus needs to affect the basal layer cells showing stem cell-like characteristics with capacity of proliferation. *Human papillomavirus* oncoproteins, mainly the E5, E6, and E7 genes, incorporate DNA viral genes inside the host (human), DNA, developing into a malignant form and leading to tumor growth. These proteins increase cell proliferation and decrease apoptosis by corrupting various intracellular signaling pathways such as the degradation of p53 and pRB tumor suppressors, alteration of cell cycle regulation, p16 overexpression, driving the S-phase reentry in the upper epithelial layers or apoptosis resistance. The overexpression of E6/E7 core genes represents an essential key that have an impact on tumor suppressor genes, especially those that regulate the cell cycle [5–7].

Low-risk HPV types do not stimulate cell proliferation and the role of E6 and E7 proteins is uncertain in infected basal layer cells because are not usually related to invasive cancer. In benign HPV infections, the HPV DNA accesses the cellular nucleus but is localized extrachromosomally, compared to invasive cancers where the viral DNA integrates inside the host genome. Regarding Lr-HPV types, the role of wound healing response in determining the initial proliferation of the HPV infected cell is believed to be crucial. Understanding the altered molecular mechanisms, which occur during the development of HPV infection to cervical intraepithelial neoplasia and invasive cervical cancer, offers a better comprehension of the various pathways involved and inspires the new development of targeted treatment via nanotechnology [5, 6, 8, 9].

The implementation of screening programs for cervical cancer among females brought an enormous positive impact in the early diagnosis and treatment. The screening tests comprise cytology test and HPV detection, which are able to detect the premalignant lesions and the serotypes of HPV. Moreover, three anti-HPV vaccines have been produced against some of the HPV types in order to decrease the incidence and prevalence of HPV still, they could not entirely remove the condition. These vaccines create antibodies for HPV serotypes 16 (the monovalent vaccine), 16, 18 and 6, 11, 16, 18, respectively [10].

Since this condition represents a wide health problem due to its increase mortality and morbidity, various treatments have been developed according to the cancer staging. In the initial stage, surgical treatment or radiotherapy is very effective. For terminal stages, guidelines recommend chemotherapy, radiotherapy, immunotherapy, and targeted therapy [11]. Chemotherapeutics drugs such as DNA-interactive agents and antimetabolites are usually combined with various cytotoxic drugs in order to increase the control efficacy. Chemotherapy, frequently associated with other therapies like radiotherapy or surgery, is indicated for curative purpose, in order to extend the patient's life or to alleviate pain symptoms of terminal stage patients. Unfortunately, none of the treatment leads to adequate results and their failure is due to the side effects and resistance mechanisms that most of the anticancer drugs carry. The common limitations of chemotherapies represent low solubility in water due to their hydrophobic character, lack of selectivity of cancerous cells that can induce a huge damage to normal cells also, and the potential to develop multidrug resistance [6, 7, 11]. Conventional chemotherapy used in cervical cancer has its limitations. Most of the agents are extracted and produced artificially from plants, and this causes low water solubility. The need to use different solvents to form the correct dose of chemotherapy enhances their toxicity. Another issue represents the potential for early damage of normal cells, apart from cancerous cells, due to their low selectivity. Moreover, the multidrug resistance effect can impair the drug delivery outside the cell by increasing the efflux pumps in the cell membrane. However, all these restrictions could be overcome by using nanotechnology via drug delivery systems. Nanotechnology represents the procedure of creating nanomaterials (nanoparticles, nanosheets, nanotubes, or nanorods) at the molecular and atomic stage, with sizes that go from 1 to 100 nm, and constitutes a promising approach to treat different types of cancers [6, 11].

**Figure 1:**
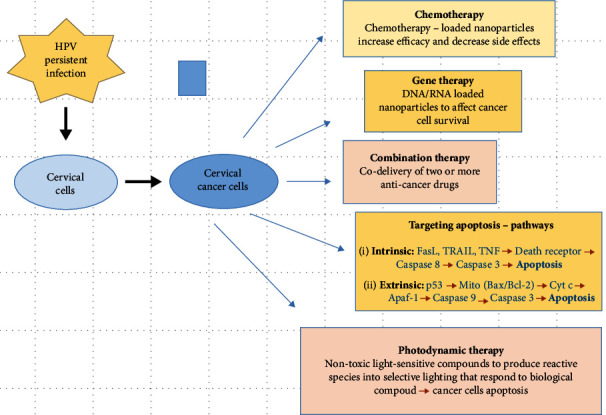
Nanotechnology application in the treatment of cervical cancer (modified after Chen et al. [17]). HPV: human papilloma virus.

Nanoscale size drugs represent the state of the art in the area of nanoparticle applications because of their amazing capacity to change important properties of drugs. The advantages of nanocarriers in cervical cancer therapy are that they can refine water solubility, agent delivery profile and diffusion, immunogenicity, and bioavailability, making them suitable for two delivery pathways: self-delivery and passive delivery. In the former, the delivery time is very important as the chemotherapeutic agent combines to the structure matter of nanocarriers in order to be easily released; otherwise, the agent will not contact the target and it will be rapidly dissociated from the carrier. In what regards the later, the agent encapsulates inside the nanocarrier due to the hydrophobic effect and will be release to the targeted site [6, 12–15]. Various treatment methods developed through nanotechnology can improve life quality and duration for patients with cervical cancer (Figure 1). Nanomaterials' ability to accumulate at the site of the tumor more than in normal cells, via a passive targeting action called the enhanced permeability and retention (EPR) effect, can improve the efficacy of the drugs, by decreasing the systemic side effects that are conventionally present. Moreover, their protective role of agents that usually degrade inside the body via different biochemical reactions and increase drug bioavailability and life. Another advantage is the possibility to combine different drugs as the nanocarrier can transport several types of agents, enhancing the potential of treatment [6, 13–16].

Among the conventional chemotherapy drugs, cisplatin presents the best response in cervical cancer treatment, being the first choice in therapeutic protocols. Cisplatin determines apoptosis of the cancer cell and deactivates its function when it binds and crosslinks with tumor DNA. However, this drug has its own limitations when side effects (neutropenia, thrombocytopenia, neurotoxicity, nephrotoxicity, or hematological toxicity) or tumor resistance occur. Various nanocarriers reduced the side effects of the drug and increase treatment feasibility and efficacy [18–22]. Paclitaxel is a chemotherapeutic drug that is widely used in human cancers, but just like cisplatin, its clinical applications are limited by drug resistance development due to various factors and dose-limiting toxicity [20, 23]. Both *in vitro* and *in vivo* studies show that curcumin increases paclitaxel-induced cytotoxicity by the downregulation of Akt pathways and nuclear factor-kB (NF-*κ*B) and enhances its efficiency in cervical cancer [23, 24].

Methotrexate (MTX) proved to be efficient in various types of tumors. This drug is an inhibitor of dihydrofolate reductase that lowers the reversal of dihydrofolate into tetrahydrofolate, which is an important key for the synthesis of DNA and RNA. A study has shown that in combination with chitosan or methoxypoly nanoparticles it is more efficient in tumor growth inhibition and proliferation [25].

Different nanoparticles have been developed to carry multiple drugs at the same time in order to increase treatment efficacy. Therefore, an *in vitro* study reveals that d-*α*-tocopheryl polyethylene glycol 1000 succinate-b-poly(*ε*-caprolactone-ranglycolide) (TPGS-b-(PCL-ran-PGA)) nanoparticles loaded with docetaxel and endostatin reduce the viability of HeLa cells and hinder the tumor growth in a xenograft model [26]. Various studies demonstrate that nanoparticles have the great potential to simultaneously carry and deliver multiple drugs [27–29], photosensitizing molecules [30–32], genetic molecules [33], and protein, enhancing the efficacy of treatment (Figure 1).

## 2. Drug Delivery Systems

**Figure 2:**
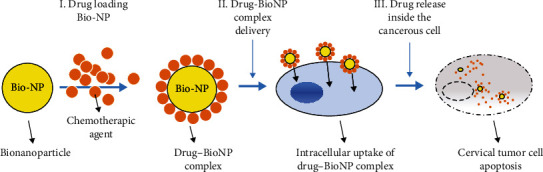
Systemic drug delivery system in cervical cancer; Bio-NP: bionanoparticles.

Drug delivery systems represent the novel pathways of drug administration that target specific situses inside the organism, in order to decrease overall toxicity and enhance bioavailability. Each drug delivery system has unique features such as physical, chemical, and morphological varieties. Moreover, particular chemical or physical interactions make them compatible with varied agent polarities. According to the type of administration, drug delivery systems are systemic and localized. Therefore, the systemic drug delivery pathways employ nanoparticles such as dendrimers, liposomes, and micelles, with specific characteristics on their surfaces that help to localize the desired situs (Figure 2) [6, 34].

**Figure 3:**
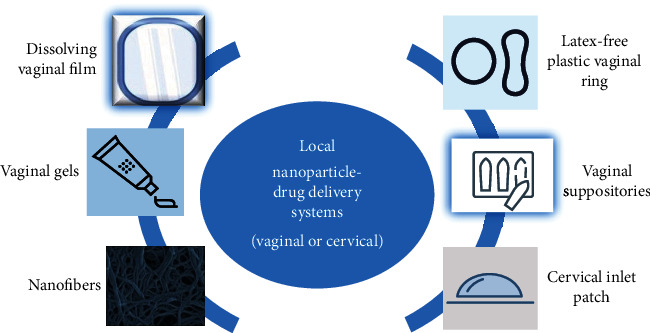
Localized drug delivery systems in cervical cancer.

Their intended role is to decrease the frequency and dosage of the agent, the systemic side effects due to their specific target, and the oscillations of drug concentration inside the body. The target specificity is achieved by nanocarrier conjugation to a variety of ligands with great affinity for the damaged cell sites such as tumor cells. Hence, the nanoparticles can encapsulate the drugs or molecules inside their structure and/or can engross the drug or molecule in the external surface. On the other side, the localized delivery pathways release outright the drug to the tumor site, limiting the drug systemic toxicity (Figure 3). The delivery system is placed near the cancer site or directly on the tumor, which is suitable for cervical cancer treatment [35–37].

## 3. Nanoparticles Used for Drug Delivery Systems

Nanoparticles applied in drug delivery systems present various advantages compared to the conventional therapy for cervical cancer. Their size is below 1000 nm so they can go through the tiniest vessels and bypass the phagocytes' rapid clearance in order to last longer in the bloodstream. In addition, they easily enter cells or tissues to reach targeted organs such as the cervix, liver, spleen, or others. Because of their biodegradability, heat sensitivity of structures, and pH, they possess controlled-release attributes which make them capable for drug or molecule delivery [6].

### 3.1. Lipid-Based Nanocarriers

Lipid nanocarriers are usually synthetized from phospholipids, triglycerides, or cholesterol, and they enhance drug solubility, encapsulation, and delivery, thus raising chemotherapy absorption. Beside lipid nanocarriers, other organic nanoparticles are liposomes, solid lipid nanoparticles (SLN), and nanostructured lipid carriers (NLC) [38, 39]. Liposomes were firstly found in 1960 by Alec Bangham and are probably the most studied among nanoparticles [40, 41]. A main advantage of liposomes is that they are able to deliver both hydrophilic and hydrophobic drugs, such as phytochemicals, chemotherapeutic particles, and immune-cytokines in order to reach the cancer cells. Recent studies, have shown their efficient applicability in cervical cancer when combined with cisplatin (lipoplatin), paclitaxel, interleukin 2 (IL-2), and curcumin. When loaded with liposomes nanoparticles increase drug stability, bioavailability, and tumor cell absorption [18, 24, 42, 43].

Solid lipid nanoparticles are advantageous because they raise drug solubility and reduce the dose of drug. Moreover, SLNs enhance drug stability by its lipid matrix that has the role to secure the components that are chemically instable and supply the attachment and incorporation inside the cancerous cell [39]. Nevertheless, SLN presents a decreased loading volume of the drug and rapid expulsion of the drug through its depositing [44]. Colloidal drug carrier systems as nanostructured lipid carriers are formed by a mix of liquid and solid lipids which makes them good candidates for drug delivery. They potentiate the bioavailability of compounds with low solubility, protect susceptible active agents, and ease the directed delivery of the drug [45, 46]. Zhang et al. used both in HeLa cells and mouse cervical cancer models, folic acid (FA) modified, cisplatin- (CIS-) loaded nanostructured lipid carriers (NLCs) for cervical cancer chemotherapy, showing its efficacy in selective release in tumor cells that highly express FA receptors. Moreover, the FA-CIS-NLC-targeted transfer CIS to cancer cell increases its antitumor power [47].

### 3.2. Polymeric and Dendrimeric Nanoparticles

Polymeric nanoparticles are biocompatible structures that have a great preservation for chemical or enzyme-catalyzed degradation, penetration capacity, and controlled release inside the cancerous cell. They are capable to load antibodies, DNA or RNA, allowing particular interaction in the individual targets [48–50]. The drug delivery is easily directed by the degradation rate of nanopolymers, making it simpler to control. The degradation products have no toxicity, and absorbable parts metabolize with no need of surgical removal intervention, once the agent delivery is depleted [51]. Various derivatives of poly(lactide-*co*-glycolide) (PGLA) loaded with docetaxel, in both *in vitro* and *in vivo* cervical cancer treatment, showed good delivery control and sustainability and revealed a higher efficiency of cellular uptake and antitumor ability [26, 52, 53]. When combined with polymeric nanoparticle and drug, the folate receptor acts as a feasible target on cervical cancer cells, due to its high expression [54, 55]. Studies showed that FA conjugated with chitosan [56], chitosan-coated PLGA nanocarriers [57], gelatin [54], and L-tyrosine-polyphosphate [58] and filled with selenocystine, carboplatin, cisplatin, and silver carbene complex present a 10-fold higher specificity of drug compared with the control.

The structure of dendrimers enables the fastening and presentation of antigen molecules on their extremity, making them multifunctional. Merkuria et al. show that doxorubicin-loaded dendrimers garnished with IL-6 antibodies display greater cellular incorporation and decrease the value of IC50. Moreover, it raises the drug loading rate and drug discharge rate and has a higher cytotoxicity when compared with RGD (arginyl-glycyl-aspartic acid) peptide-conjugated in HeLa cervical cancer cells [59].

### 3.3. Carbon-Based Nanoparticles

Carbon-based nanotubes have been extensively studied since 1990 and are attractive nanoparticles in increasing the pharmacological profile of various diagnosis and therapeutic agents. Nowadays, they divide into single-walled carbon nanotubes (SWCNT) and multiple-walled carbon nanotubes (MWCNT), each with different characteristics. They brought great contribution in imaging and drug delivery, due to their thermal, mechanical, and electrical features [60, 61]. The photothermic treatment of solid tumors using SWCNT enhanced by near-infrared light (NIR) determine a noninvasive cell death, without noxious side effect. The efficiency of SWCNT and MWCNT was proven in the treatment and early diagnosis of cervical cancer, but even if they are very promising, there are still some issues regarding toxicity and biocompatibility due to the lack of selectivity for these treatments [59, 62].

### 3.4. Metallic Nanoparticles (MNP)

The synthesis of MNP (gold, silver, iron oxide, and silica) is achieved by chemical and physical procedures. Compared to other nanoparticles, gold and silver nanocarriers possess a particular feature, called the surface plasmon resonance (SPR), which makes the cellular surface functionality more versatile and biocompatible. There are still doubts regarding their toxicity related to the ionized or the particulate structure. Two mechanisms were proposed, the transcytosis and paracellular conveyance, but the *in vivo* carriage and the absorption process are still unclear [63, 64]. However, gold nanoparticle- (AuNP-) loaded gallic acid (GA) slows down tumor cells proliferation by causing cellular apoptosis in CaSki or HeLa cell cultures, compared to free GA. Surprisingly, a high dose of AuNPs-GA (150 *μ*M) complex did not affect the normal cervical cells, compared to the GA group. Therefore, the study revealed that even if AuNPs-GA efficiency is lesser than GA alone, no cellular toxicity was reported in the normal cervical cells group when AuNPs-GA was applied [65]. Another study shows that AuNP-conjugated doxorubicin presents a higher anticancer activity in human cervical cancer cells, compared to free drugs [66]. The AuNP conjugation with bioactive molecules reduces overall toxicity and increases mitochondria targeting in cancer cells. Phloroglucinol conjugated with AuNPs determines apoptosis in HeLa cancer cells by increasing the permeability of the mitochondrial membrane [67]. Another study showed that if loaded with *Podophyllum hexandrum* plant extract, the AuNPs determine DNA impairment and cellular cycle block at G2/M phase in HeLa cells [68].

Bionanotechnology via green synthesis is safer and less expensive. The photosynthesized *Catharanthus roseus* (CR) AuNPs enhance mitochondrial-mediated apoptotic signaling pathway through reactive oxygen species (ROS), causing high toxicity in HeLa cell cultures [69]. Silver nanoparticles (AgNPs) have an extensive use in the health care industry due to their particular features, being an anti-inflammatory, antibacterial, antiviral, antiangiogenic, or anticancer product. If silver is used in low quantities, it causes no damage in animal cells, compared to increased toxicity against bacteria or cancerous cells. In cervical cancer treatment, few studies are available for AgNPs obtained by chemical synthesis like ultraviolet radiation, photochemical reduction, laser ablation, or aerosol technologies [70–72]. Just like in the case of AuNPs, green synthesis of AgNPs is mostly used due to ecofriendly production. Yuan et al. reported that AgNPs conjugated with camptothecin (CPT) showed cell proliferation inhibition and enhanced cytotoxicity and apoptosis, through varied mechanisms such as raising the levels of oxidative stress markers and accelerating multiple proapoptotic gene expression [73]. Varied medicinal plants with antioxidant properties such as *Piper longum* [74] and *Cleistanthus collinus* [75] have been used for AgNP synthesis, showing favorable responses in anticancer treatment.

Iron oxide nanoparticles have been extensively studied due to their amazing capacity of combining drug delivery systems, imaging, and treatment characteristics. The effect of supermagnetic DMSO@ *γ*-Fe₂O₃, combined with chemotherapy agent carmustine on cervical cancer under a variable magnetic field showed an increased toxic effect, enhanced by nanomagnetic fluid thermotherapy used on cervical cells. Superparamagnetic iron oxide nanoparticles (SPIONs) are great for their noninvasive diagnosis and therapeutic use but there is still a slowly progress into clinical application [76, 77].

Mesoporous silica nanoparticles (MSNs) present great features such as tunable proportion, high load volume, morphology, stability, and simply possibility to modify internal and external surfaces of the NP and this make them very attractive for the cervical cancer diagnosis, treatment, and promising in cancer theragnostics [78]. Using MSNs, Franco et al. showed increased cellular link-up and nanomaterial transfer among immune cells and augmentation of interaction between MSNs and macrophages to coordinate an immune response in cervical cancer [79].

Selenium nanoparticles (Se NP) have demonstrated their use in cervical cancer, and their antitumor outcome is due to the inhibition of migration and invasion activity which offer an antimetastatic effect [29]. Rajkumar et al. analyzed the anticancer properties of green synthesis Se NP from *Pseudomonas stutzeri* (MH191156) as an efficient source of Se NP and its antitumor and antiangiogenic characteristics against cervical cancer cells [80]. Other authors proved its efficacy in cervical cancer by using it as a drug delivery system combined with doxorubicin [81], or by targeted siRNA delivery silence Derlin1, enhancing anticancer effect [82].

### 3.5. Inorganic Nanoparticles

The cooper oxide nanoparticles revealed amazing cytotoxicity results when they were tested against different cancer cells like human cervical (HeLa cells), breast (MCF-7 cells), lung (A549 cells), and epithelioma (Hep-2 cells) [83]. Although zinc oxide nanoparticles have been successfully used in the cosmetics industry due to their photocatalytic action, it was shown that if used among paclitaxel and cisplatin determine selective cancerous cell death *in vitro* human squamous cell carcinoma [84]. Barium carbonate like AuNP is used through the green synthesis of NP and can generate tumor cell apoptosis, by affecting the size and surface activity of the cell and increasing the production of reactive oxygen species (ROS) [85, 86]. Magnetic nanoparticles are made of a nanomagnetic material which has magnetic response and high paramagnetism. The magnetic nanoparticles that are usually used are magnetite and maghemite. Due to their properties, they can be placed under a magnetic field in order to deliver a targeted drug or as a magnetic resonance imaging contrast agent [87].

### 3.6. Micellar Nanocarriers

Micellar nanocarriers represent colloidal particles formed by amphiphilic block copolymers, which can combine among them. *In vivo*, they are very stable, with the capacity to solubilize water-hydrophobic drugs and increase the blood-circulating period due to their small sizes that vary between 10 and 100 nm [88]. When their surface is PEGylated, they can cross via passive transport through inflammatory tissues and tumor vessels and maintain a higher treatment concentration in the tumor site. There are three conjugation methods between drugs and copolymers by which micelles forms. The direct dissolution process employs the water environment to load the drug with the polymeric micelles. The solvent evaporation method uses a volatile organic solvent to disband the desired drug and copolymer. The third process is dialysis, in which through a dialysis bag the agent, washed up in solution, and the copolymer soaked in organic solvent, are combined. The micelles form subsequent dialysis process of the two components [89–91].

### 3.7. Protein and Polysaccharide Nanocarriers

Proteins are natural biomolecules intensely used in nanotechnology with single or multiple functions. In order to increase the targeting process, the protein nanocarrier is damaged by chemical alteration and after that it is conjugated with the targeting ligand, which will amplify the exact delivery toward cells or tissues. Albumin is a multifunctional protein that contains some hydrophobic pockets, which ease the link between the drug and amphiphilic or hydrophobic molecules. Li et al. used in a phase 2 study nanoparticle albumin-bound paclitaxel (nab-paclixatel) and nedaplatin (NDP) for patients with advanced, recurrent, and metastatic cervical cancer with good activity and tolerable results, [92]. Alberts et al. showed similar results in a phase 2 trial, using albumin-bound nab-paclitaxel in the treatment for recurrent and metastatic cervical cancer [93].

Gelatin nanoparticles (GNP) have a large applicability to target damaged tissue such as cancer, tuberculosis, vasospasm, or HIV [94]. The polysaccharides are much the same as proteins, being composed of monosaccharides clusters connected by O-glicosidic bonds. They are advantageous because they are very versatile and have specific attributes. Due to their similar structure with extracellular matrix they can bypass various immunological reactions, making them suitable candidates for drug delivery systems [95]. Still, the polysaccharides can easily disintegrate (oxidation process) if melting temperature is used for their achievement. Moreover, features such as water solubility put limits on some applications areas [96].

## 4. Future Perspectives

Regarding the theoretical models of controlled drug release, in addition to the classical ones, which invoke diffusion equations by Fickian, and non-Fickian processes, a new class of model is based on the description of drug release processes by continuous and indistinguishable curves (multifractals curves). As the use of such curves implies the property of self-similarity in any release points of the matrix (i.e., the part reflects the whole, the whole reflects the part, i.e.,the holographic principle), it follows that drug delivery mechanisms can be assimilated to holographic implementations of release dynamics. Following this, the class of holographic mechanism of controlled drug release it may be proposed [97–104].

**Figure 4:**
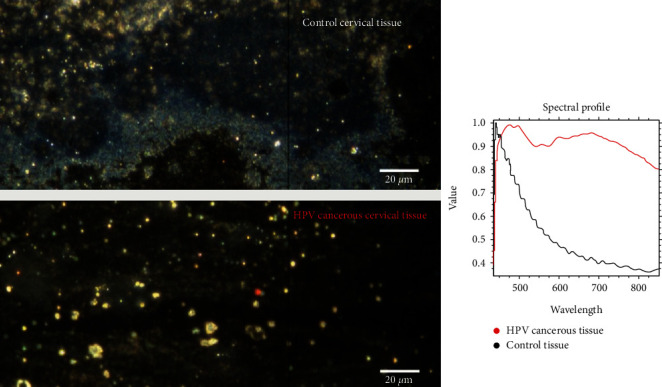
Enhanced dark field hyperspectral microscopy-control cervical tissue versus HPV 16 cervical tissue.

For a deep understanding of phenomena, which happened in the human organism, in cases with human papilloma virus infection, we decided to analyze by comparison of two samples (HPV 16 and control) on the micronic scale. Moreover, after this achievement of obtaining the nanoscale optical imaging of the samples on 20 *μ*m through the enhanced darkfield hyperspectral microscopy, we realized a spectral analysis (see Figure 4). The interpretation of the obtained spectral profile showed that there is a clear difference between the two samples. Thus, the graph corresponding to the control sample is defined by a decreasing nondifferential curve that shows an exponential decrease. In contrast, the graph corresponding to the HPV sample is represented by an increasing nondifferential curve showing a saturation level. In addition, for any of the nondifferential curves presented above, fractal dimensions can be calculated, as well as their succulence and lacunarity.

With this occasion, in cases of patients with HPV infection, more perspectives can be opened on the main directions within artificial intelligence that can become a primary step for much faster identification and diagnosis compared to Pap smear tests or DNA HPV test. This new technique will have at least the same accuracy.

The dimension, configuration, and some particular chemical and biophysical features concur to nanoparticle efficiency. Moreover, the specific biochemical and biophysical characteristics that a drug possess bring a huge contribution to the perfect nanoparticle-drug delivery complex. Further modulation regarding the dimension, shape, surface feature, and aqueous-solubility may augment nanoparticles bioavailability and bioactivity. Even though the nanotechnology is continuously changing nowadays, there are still some doubts regarding practical applications of nanoagents. Several questions regarding their safety, toxicity, and effective regulation need to be answered. Because of the toxic reactions that nanoparticles may have in normal cells, recently, there have been attempts to conjugate nanoparticles with natural compounds via the green chemistry pathway. Various studies showed that biosynthetic processes through bionanotechnology reduce the toxicity dilemma and lower the side effects that most of the conventional nanoparticles have [105]. For this reason, hybrid nanocarriers represent the most encouraging application for nanomedicine, giving the heterogeneous properties of various compounds in a singular delivery system.

## 5. Conclusions

It is clear that nanoparticles represent an important key for the progress of drug delivery systems in cervical cancer, having an extensive use in prevention, screening, diagnostic, management, and treatment compared to other methods. All the assembled data from the literature show that chemotherapy drug-loaded nanocarriers provide a pertinent therapeutic strategy against cervical cancer, and the ongoing perfection of drug delivery systems will further integrate nanotechnology into clinical practice. As any hologram function as a deep learning process, the artificial intelligence can be proposed as a new analysis method of cervical cancer.

## Data Availability

The data used to support the findings of this study are included within the article.
